# Supplementary material for: A comprehensive evaluation of self-attention for detecting regulatory feature interactions
Source: NAR Genom Bioinform. 2026 Jan 6;8(1):lqaf209. doi: 10.1093/nargab/lqaf209 (PMC12770979; doi:10.1093/nargab/lqaf209)
Supplement: lqaf209_Supplemental_File [file lqaf209_supplemental_file.pdf]

A Comprehensive Evaluation of Self Attention for Detecting Regulatory Feature Interactions: Supplemantary Material

SUPPLEMENTARY FIGURES

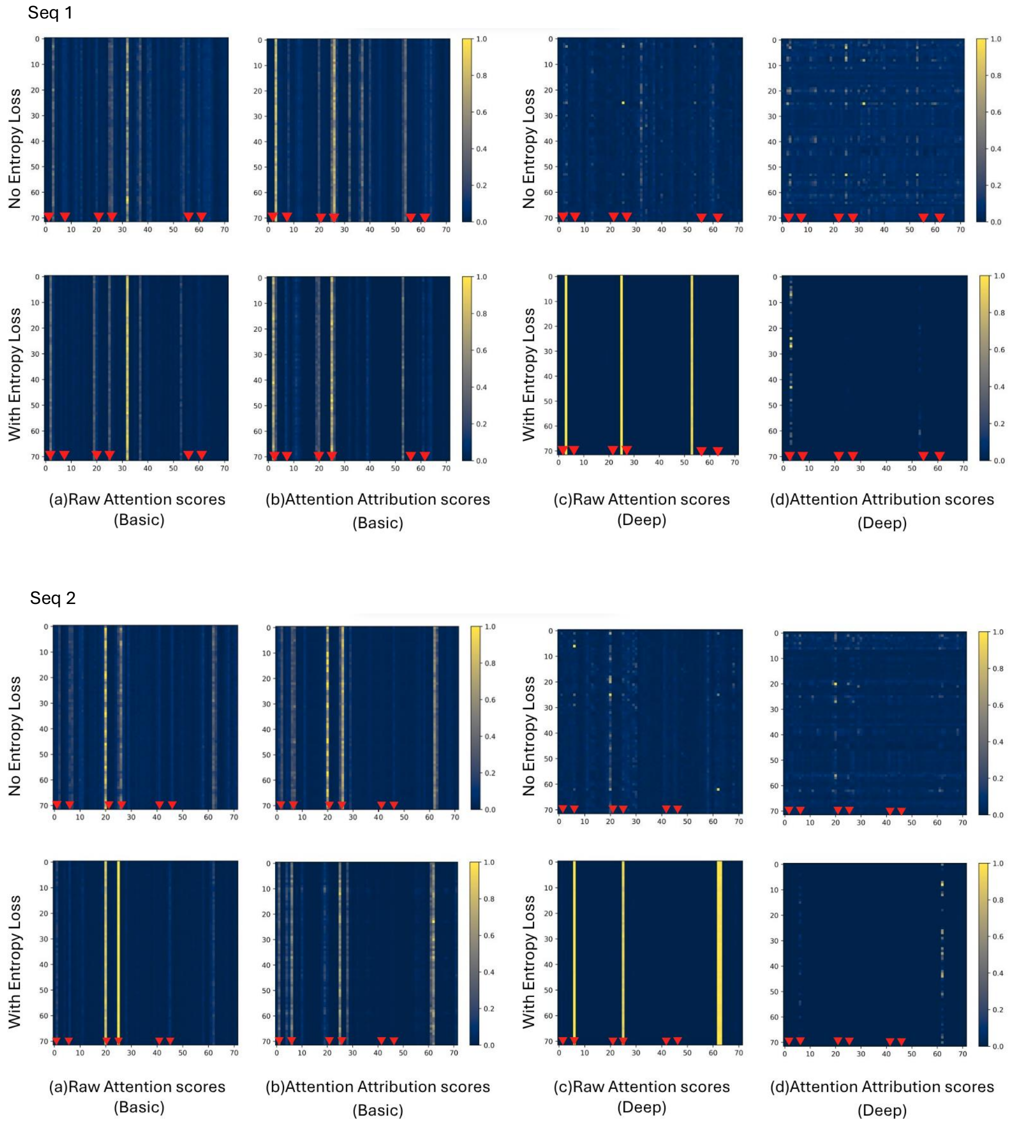

Fig. S1. Heatmaps of attention values. These are shown for four true positive test sequences from Data-40, two sequences in this figure, and two additional sequences in Fig. S2. The top row for each sequence represents models trained without the entropy loss, while the bottom row shows models trained with entropy loss. Columns (a) and (c) were computed based on raw attention values, while (b) and (d) represent attention attribution. Columns (a) and (b) are from the Basic architecture, whereas (c) and (d) illustrate the Deep architecture. In all heatmaps, both axes represent positions within the sequence after max-pooling. Each position on the y-axis corresponds to attention probabilities with respect to each position on the x-axis. This visualization enables comparison of attention patterns across different model architectures, training strategies, and score types, highlighting the impact of the entropy loss and architecture depth. Red arrows indicate the positions where motifs are embedded, adjusted for the size of the pooling window.

Seq 3

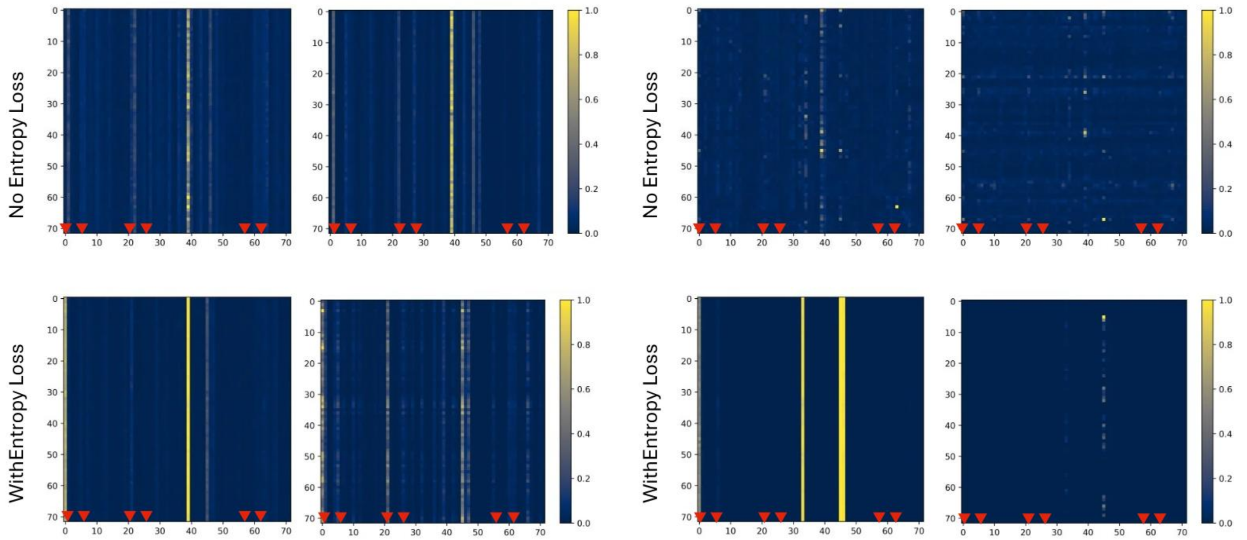

(a)Raw Attention scores (Basic) | (b)Attention Attribution scores (Basic) | (c)Raw Attention scores (Deep) | (d)Attention Attribution scores (Deep)

Seq 4

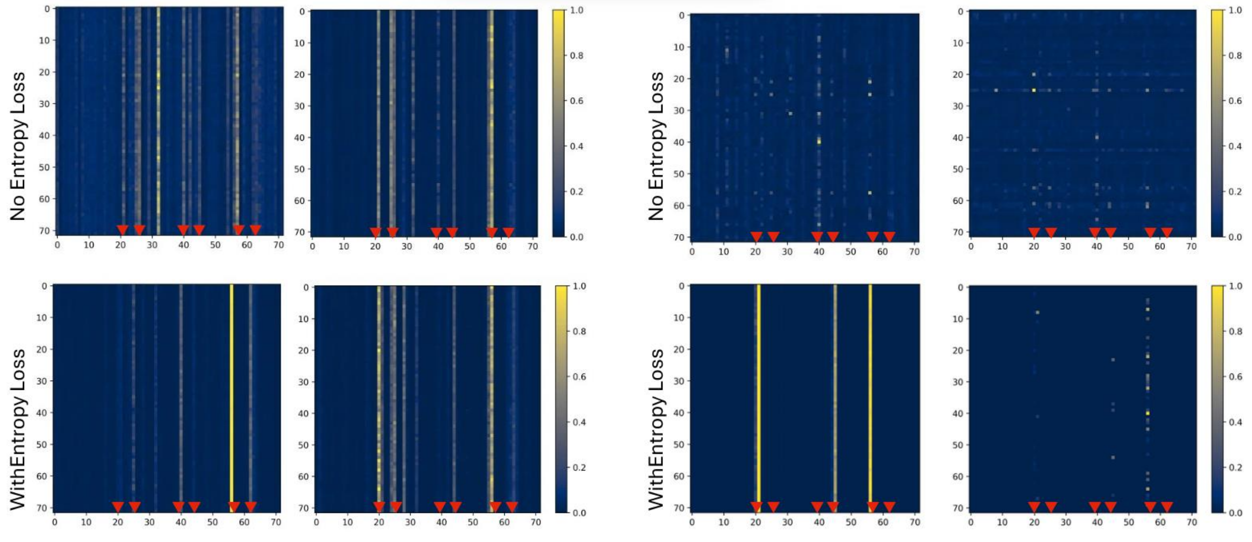

(a)Raw Attention scores (Basic) | (b)Attention Attribution scores (Basic) | (c)Raw Attention scores (Deep) | (d)Attention Attribution scores (Deep)

Fig. S2. Heatmaps of attention values. See caption of Fig. S1 for details.

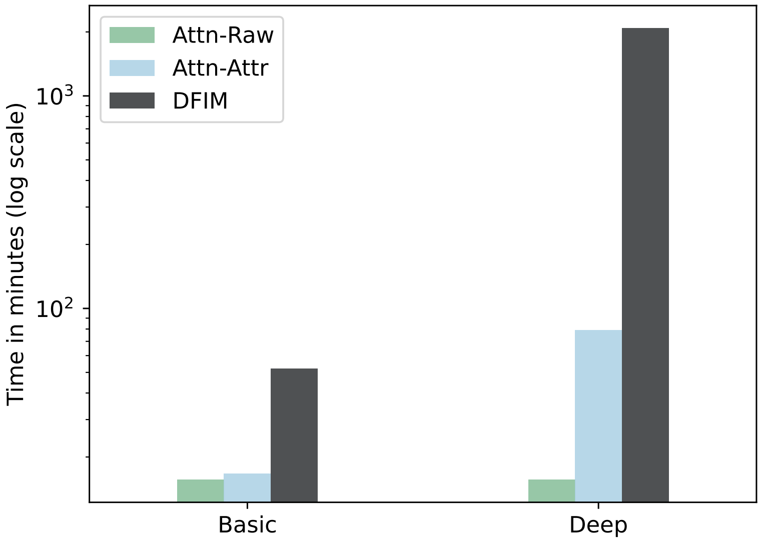

Fig. S3. Average runtime for SATORI with raw attention scores, attention attribution scores, and DFIM to estimate interactions for Data-40.

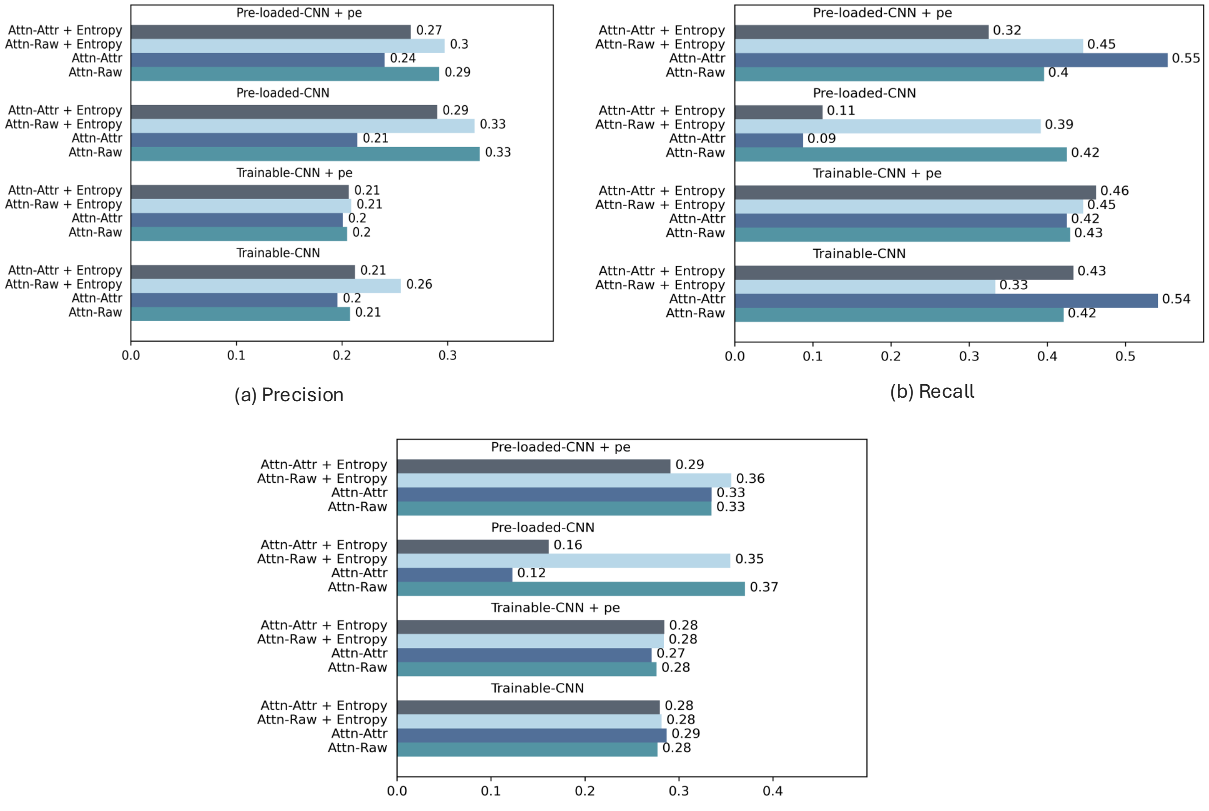

(c) F1-score

Fig. S4. Comparative performance of the SATORI-Basic model on simulated datasets with longer sequences (1500 bp) containing 80 motif pairs. We report interpretation performance as: Precision (a), Recall (b) and F1-score (c). Results are shown for eight model variants: SATORI-Basic with trainable or pre-loaded CNN filters, each trained with or without entropy loss, and with or without positional encoding. Performance metrics are averaged over three dataset instances and reported using both raw attention scores (Attn-Raw) and attention attribution scores (Attn-Attr).

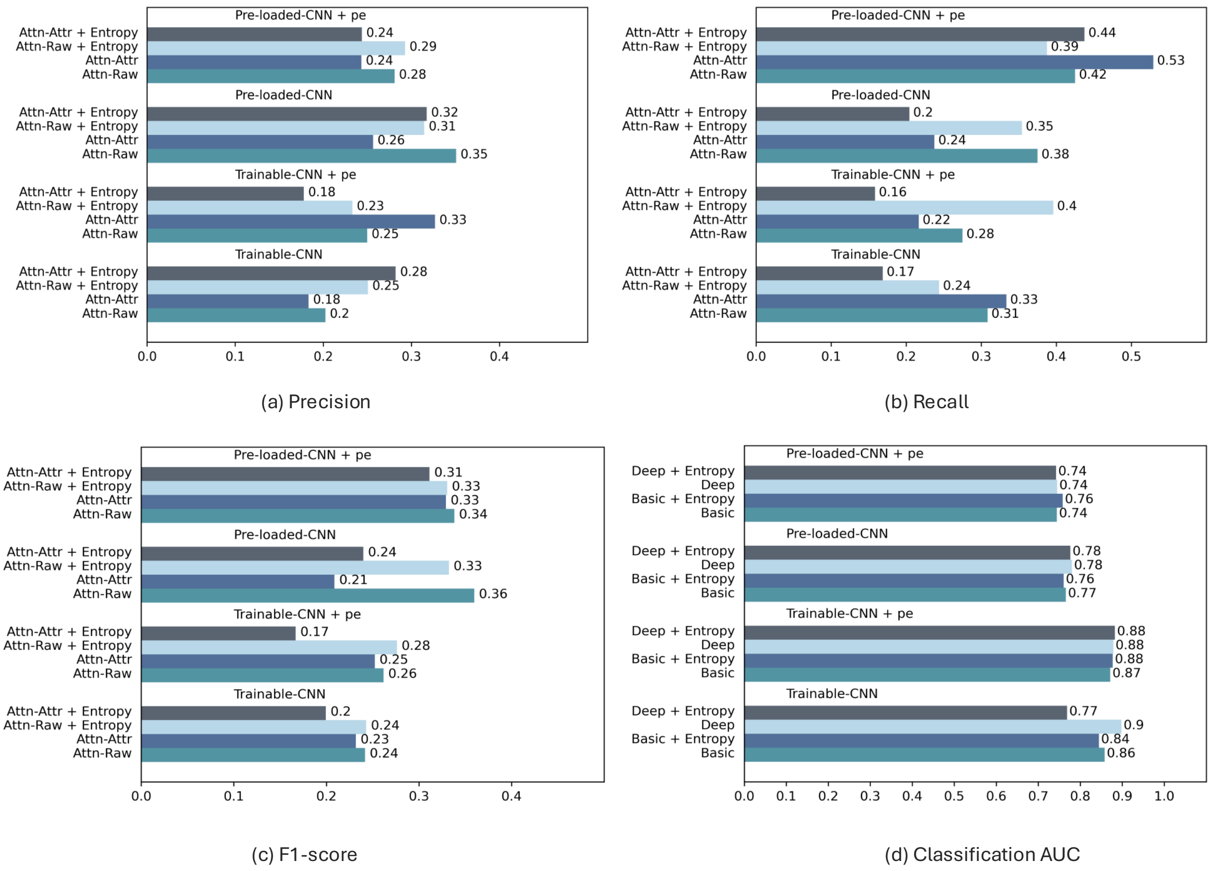

(a) Precision

(b) Recall

(c) F1-score

(d) Classification AUC

Fig. S5. Comparative performance of the SATORI-Deep model on simulated datasets with longer sequences (1500 bp) containing 80 motif pairs. We report interpretation performance as: Precision (a), Recall (b) F1-score (c) and classification AUC (d). Results are shown for eight model variants: Deep-SATORI with trainable or pre-loaded CNN filters, each trained with or without entropy loss, and with or without positional encoding. Performance metrics are averaged over three dataset instances and reported using both raw attention scores (Attn-Raw) and attention attribution scores (Attn-Attr).

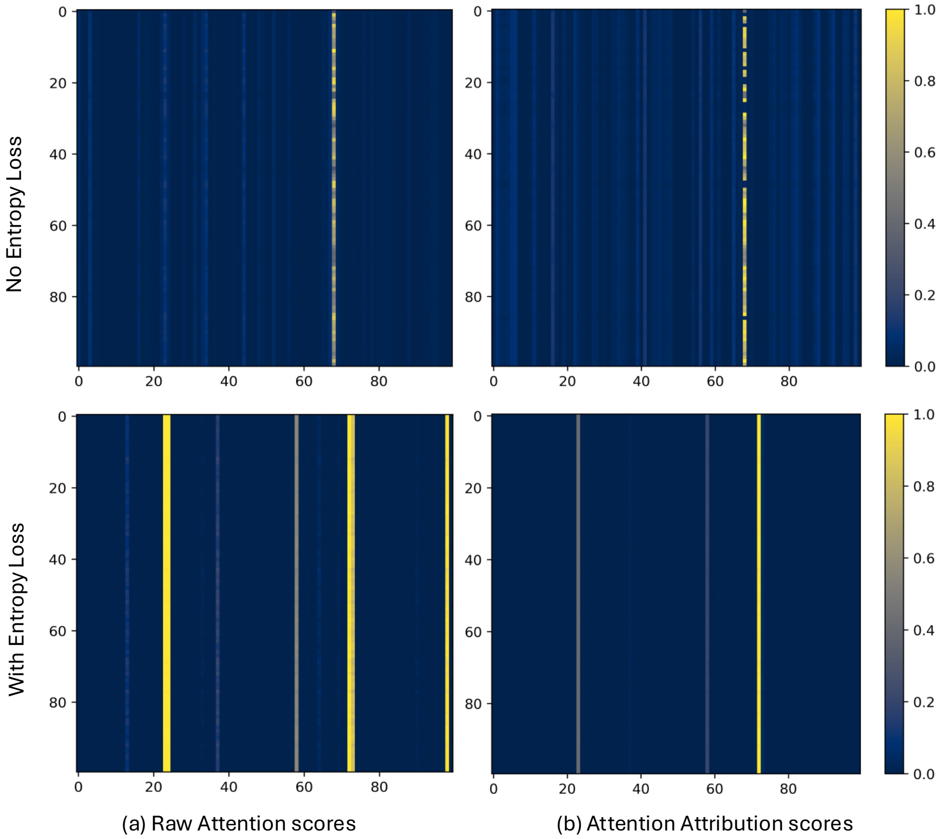

Fig. S6. Heatmaps comparing raw attention scores in column (a) and attention attribution scores in column (b) for a true positive test sequence from the Human Promoters dataset using the Basic architecture. The top row shows attention scores without the entropy loss, and the bottom row comes from a model that uses the entropy loss.

SUPPLEMENTARY TABLES

Table S1. Basic SATORI architecture's hyper-parameters, search space and sampling method

Hyper-parameters	Search space	Sampling
No. of CNN filters	[200, 300, 400]	Uniform
CNN Filter Size	[13, 15, 17, 19]	Uniform
No. of heads	[2, 4, 8]	Uniform
Head Size	[32, 64, 128]	Uniform
FC Layer Size	[100, 200, 256,512]	Uniform
No of FC layers	[1,2,4]	Uniform
Layer Normalization	[Yes, No]	Uniform
Optimizer	[SGD, Adagrad, Adam]	Uniform
Learning Rate	[0.0001,0.1]	Log uniform
Momentum (SGD)	[0.95,0.99]	Sqrt uniform
Weight Decay	$[10^{-6}, 10^{-1}]$	Log uniform
Batch Size	[32, 64, 128, 256]	Uniform
No of epochs	[30, 40, 50, 60]	Uniform
Intialization Seed	[0,1,2]	Evaluate all

Table S2. Architecture specifications of basic and deep SATORI models for simulated and real datasets

Architecture specs		Basic (Simulated)	Deep (Simulated)	Basic (Real dataset)	Deep (Real dataset)
Conv1d	# of filters	200	256	200	256
	Filter size	13	13	13	13
	Padding	0	0	6	6
Batch Normalization & ReLU()	Same for all				
MaxPool1d	Filter size	4	4	6	6
	Padding	0	0	6	6
Dropout	0.2				
Multihead Attention	Head size	64	64	32	32
	# of heads	4	4	8	8
LayerNorm		Yes	No	No	No
Linear 1	Size	512	512	1024	1024
ReLU() & Dropout	0.2				
Linear 2	Size	100	**Supplementary Layers Block**	-	**Supplementary Layers Block**
ReLU () & Dropout		0.2		-	
Readout	Normalize				
Linear 3	Size	**2**		**No of classes**	

Table S3. SEI-inspired Supplementary Layers Block

**Layer**	**# of filters**	**Filter size**	**Padding**	**Dilation**
Conv1d	480	9	4	-
Conv1d	480	9	4	-
Conv1d	480	9	4	-
ReLU()				
Conv1d	480	9	4	-
ReLU()				
Dropout(0.4)				
Dconv1d	480	5	4	2
ReLU()				
Dropout (0.4)				
Dconv1d	480	5	8	4
ReLU()				
**Output Layer**				

Table S4. Number of matched interactions inferred by different methods with HIPPIE databases

**Methods**	**Matches in HIPPIE**
DFIM	3
Attn-Raw	1
Attn-Raw + Entropy	3
Attn-Raw[Pre-loaded CNN] + Entropy	30
Attn-Attr	2
Attn-Attr + Entropy	4
Attn-Attr[Pre-loaded CNN] + Entropy	27
Attn-Raw[Deep]	5
Attn-Raw + Entropy[Deep]	4
Attn-Raw[Pre-loaded CNN] + Entropy[Deep]	21
Attn-Attr[Deep]	0
Attn-Attr + Entropy[Deep]	4
Attn-Attr[Pre-loaded CNN] + Entropy[Deep]	25

Table S5. Significant matches with HIPPIE database inferred by SATORI basic using Attn-Raw scores on Human Promoters Dataset

TF Interaction	TF1 Family	TF2 Family	P-value
SP7⟷KDM2B	C2H2 ZF	CxxC	3.50×10^{-5}

Table S6. Significant matches with HIPPIE database inferred by SATORI basic using Attn-Attr scores on Human Promoters Dataset

TF Interaction	TF1 Family	TF2 Family	P-value
KDM2B⟷SP7	CxxC	C2H2 ZF	9.00×10^{-4}
ZFY⟷ZFX	C2H2 ZF	C2H2 ZF	5.00×10^{-5}

Table S7. Significant matches with HIPPIE database inferred by SATORI basic using DFIM on Human Promoters Dataset

TF Interaction	TF1 Family	TF2 Family	P-value
TLX2⟷KDM2B	Homeodomain	CxxC	8.71×10^{-4}
KDM2B⟷SP7	CxxC	C2H2 ZF	5.56×10^{-4}
ZFY⟷ZFX	C2H2 ZF	C2H2 ZF	4.90×10^{-3}

Table S8. Significant matches with HIPPIE database inferred by SATORI basic with Entropy Loss using Attn-Raw scores on Human Promoters Dataset

TF Interaction	TF1 Family	TF2 Family	P-value
SP7⟷KDM2B	C2H2 ZF	CxxC	8.95×10^{-3}
SP7⟷SIX4	C2H2 ZF	Homeodomain	6.56×10^{-3}
TLX2⟷KDM2B	Homeodomain	CxxC	9.24×10^{-3}

Table S9. Significant matches with HIPPIE database inferred by SATORI basic with Entropy Loss using Attn-Attr scores on Human Promoters Dataset

TF Interaction	TF1 Family	TF2 Family	P-value
SP7⟷KDM2B	C2H2 ZF	CxxC	1.65×10^{-4}
SP7⟷SIX4	C2H2 ZF	Homeodomain	2.99×10^{-2}
ZFX⟷ZFY	C2H2 ZF	C2H2 ZF	7.80×10^{-11}
TLX2⟷KDM2B	Homeodomain	CxxC	5.01×10^{-3}

Table S10. Significant matches with HIPPIE database inferred by SATORI basic with Pre-loaded CNN and Entropy Loss using Attn-Raw scores on Human Promoters Dataset

TF Interaction	TF1 Family	TF2 Family	P-value
PPARA, RARA, RARB⟷ESR1	Nuclear receptor	Nuclear receptor	3.69×10^{-2}
PPARA, RARA, RARB⟷ESR2, ESRRA	Nuclear receptor	Nuclear receptor	3.69×10^{-2}
MECP2⟷PPARG	MBD	Nuclear receptor	4.13×10^{-3}
NR0B1⟷ESR1	Unknown	Nuclear receptor	4.13×10^{-3}
NR0B1⟷PPARG	Unknown	Nuclear receptor	8.76×10^{-3}
ID2, NPAS2, MLXIP, MYC, MNT⟷TFDP1	bHLH	DP,E2F	8.76×10^{-3}
EPAS1⟷USF2	bHLH	bHLH	3.69×10^{-2}
ERF, ETV1, ELK1, ETV3⟷NR2C2	Ets	Nuclear receptor	1.80×10^{-2}
NR2C1⟷NR2C2	Nuclear receptor	Nuclear receptor	3.69×10^{-2}
SP2, SP1⟷NR2C2	C2H2 ZF	Nuclear receptor	3.69×10^{-6}
SP2, SP1⟷ETS1, SMARCC2	C2H2 ZF	Ets, Myb/SANT	9.57×10^{-4}
SP2, SP1⟷REST	C2H2 ZF	C2H2 ZF	3.69×10^{-2}
SP5, KLF12, KLF7⟷NR2C2	C2H2 ZF	Nuclear receptor	9.42×10^{-8}
JUN⟷PURA	bZIP	Unknown	3.69×10^{-2}
WT1⟷NR2C2	C2H2 ZF	Nuclear receptor	1.32×10^{-5}
SMAD2⟷ESR2, ESRRA	SMAD	Nuclear receptor	4.13×10^{-3}
TGIF1⟷SMAD1	Homeodomain	SMAD	3.69×10^{-2}
SMAD3, SMAD4⟷PPARG	SMAD	Nuclear receptor	4.13×10^{-3}
BRCA1, ZBTB33⟷E2F1	C2H2 ZF, EIN3	E2F	1.80×10^{-2}
RFX4⟷RFX2	RFX	RFX	8.76×10^{-3}
THRA, RXRB⟷NR2C2	Nuclear receptor	Nuclear receptor	2.02×10^{-3}
ESR1⟷SP3	Nuclear receptor	C2H2 ZF	8.76×10^{-3}
PPARG⟷ZBTB3	Nuclear receptor	C2H2 ZF	2.29×10^{-4}
NR2C2⟷MAF, CIC, NRL	Nuclear receptor	Sox, bZIP	1.32×10^{-5}
NR2C2⟷SP8, KLF14, KLF16	Nuclear receptor	C2H2 ZF	2.29×10^{-4}
NR2C2⟷PAX8	Nuclear receptor	Paired box	3.69×10^{-2}
TFAP4⟷CTCF	bHLH	C2H2 ZF	1.80×10^{-2}
ETS1, SMARCC2⟷KDM2B	Ets, Myb/SANT	CxxC	5.19×10^{-5}
ETS1, SMARCC2⟷ETS2	Ets, Myb/SANT	Ets	4.73×10^{-4}
PURA⟷ESR2, ESRRA	Unknown	Nuclear receptor	8.76×10^{-3}

Table S11. Significant matches with HIPPIE database inferred by SATORI basic with Pre-loaded CNN and Entropy Loss using Attn-Attr scores on Human Promoters Dataset

TF Interaction	TF1 Family	TF2 Family	P-value
MECP2⟷PPARG	MBD	Nuclear receptor	4.69×10^{-3}
NR0B1⟷ESR1	Unknown	Nuclear receptor	1.01×10^{-2}
NR0B1⟷PPARG	Unknown	Nuclear receptor	2.08×10^{-2}
ID2, NPAS2, MLXIP, MYC, MNT⟷TFDP1	bHLH	DP,E2F	1.01×10^{-2}
EPAS1⟷USF2	bHLH	bHLH	4.33×10^{-2}
ERF, ETV1, ELK1, ETV3⟷NR2C2	Ets	Nuclear receptor	2.08×10^{-2}
NR2C1⟷NR2C2	Nuclear receptor	Nuclear receptor	4.33×10^{-2}
SP2, SP1⟷NR2C2	C2H2 ZF	Nuclear receptor	4.82×10^{-6}
SP2, SP1⟷ETS1, SMARCC2	C2H2 ZF	Ets, Myb/SANT	1.13×10^{-3}
SP2, SP1⟷REST	C2H2 ZF	C2H2 ZF	4.33×10^{-2}
NFIA, NFIB, NFIX⟷SP8, KLF14, KLF16	SMAD	C2H2 ZF	4.33×10^{-2}
SP5, KLF12, KLF7⟷NR2C2	C2H2 ZF	Nuclear receptor	1.17×10^{-7}
JUN⟷PURA	bZIP	Unknown	1.01×10^{-2}
WT1⟷NR2C2	C2H2 ZF	Nuclear receptor	3.09×10^{-5}
SMAD2⟷ESR2, ESRRA	SMAD	Nuclear receptor	4.69×10^{-3}
TGIF1⟷SMAD1	Homeodomain	SMAD	4.33×10^{-2}
SMAD3, SMAD4⟷PPARG	SMAD	Nuclear receptor	1.01×10^{-2}
RFX4⟷RFX2	RFX	RFX	1.01×10^{-2}
THRA, RXRB⟷NR2C2	Nuclear receptor	Nuclear receptor	2.33×10^{-3}
PPARG⟷ZBTB3	Nuclear receptor	C2H2 ZF	5.54×10^{-4}
NR2C2⟷MAF, CIC, NRL	Nuclear receptor	Sox, bZIP	3.09×10^{-5}
NR2C2⟷SP8, KLF14, KLF16	Nuclear receptor	C2H2 ZF	2.79×10^{-4}
NR2C2⟷PAX8	Nuclear receptor	Paired box	4.33×10^{-2}
TFAP4⟷CTCF	bHLH	C2H2 ZF	2.08×10^{-2}
ETS1, SMARCC2⟷KDM2B	Ets, Myb/SANT	CxxC	6.33×10^{-5}
ETS1, SMARCC2⟷ETS2	Ets, Myb/SANT	Ets	5.54×10^{-4}
PURA⟷ESR2, ESRRA	Unknown	Nuclear receptor	4.69×10^{-3}

Table S12. Significant matches with HIPPIE database inferred by SATORI deep using Attn-Raw scores on Human Promoters Dataset

TF Interactions	TF1 Family	TF2 Family	P-value
TLX2⟷KDM2B	Homeodomain	CxxC	6.13×10^{-3}
SP7⟷KDM2B	C2H2 ZF	CxxC	4.58×10^{-4}
SP7⟷SIX4	C2H2 ZF	Homeodomain	5.79×10^{-3}
KDM2B⟷ESR1	CxxC	Nuclear receptor	6.52×10^{-3}
ESR1⟷ZHX1	Nuclear receptor	Homeodomain	2.06×10^{-3}

Table S13. Significant matches with HIPPIE database inferred by SATORI deep with Entropy Loss using Attn-Raw scores on Human Promoters Dataset

TF Interactions	TF1 Family	TF2 Family	P-value
SP7⟷FUBP1	C2H2 ZF	Unknown	3.77×10^{-3}
SP7⟷KDM2B	C2H2 ZF	CxxC	7.29×10^{-3}
FOXP1⟷SP7	Forkhead	C2H2 ZF	6.60×10^{-4}
ZFY⟷ZFX	C2H2 ZF	C2H2 ZF	2.75×10^{-4}

Table S14. Significant matches with HIPPIE database inferred by SATORI deep with Entropy Loss using Attn-Attr scores on Human Promoters Dataset

TF Interactions	TF1 Family	TF2 Family	Adjusted P-value
FOXP1⟷SP7	Forkhead	C2H2 ZF	3.80×10^{-3}
SP7⟷KDM2B	C2H2 ZF	CxxC	2.28×10^{-5}
SP7⟷FUBP1	C2H2 ZF	Unknown	4.10×10^{-3}
ZFY⟷ZFX	C2H2 ZF	C2H2 ZF	1.35×10^{-6}

Table S15. Significant matches with HIPPIE database inferred by SATORI deep with Pre-loaded CNN and Entropy Loss using Attn-Raw scores on Human Promoters Dataset

**TF Interaction**	**TF1 Family**	**TF2 Family**	**P-value**
MBD2⟷ESR1	MBD	Nuclear receptor	4.27×10^{-3}
NR0B1⟷ESR1	Unknown	Nuclear receptor	4.27×10^{-3}
SP2, SP1⟷ESR1	C2H2 ZF	Nuclear receptor	4.27×10^{-3}
SP2, SP1⟷ETS1, SMARCC2	C2H2 ZF	Ets, Myb/SANT	1.05×10^{-3}
NFIA, NFIB, NFIX⟷SMAD2	SMAD	SMAD	3.61×10^{-2}
JUN⟷PURA	bZIP	Unknown	1.77×10^{-2}
WT1⟷NR2C2	C2H2 ZF	Nuclear receptor	1.08×10^{-4}
SMAD2⟷ESR1	SMAD	Nuclear receptor	3.61×10^{-2}
SMAD3, SMAD4⟷ETS1, SMARCC2	SMAD	Ets, Myb/SANT	8.59×10^{-3}
SMAD3, SMAD4⟷CTCF	SMAD	C2H2 ZF	1.05×10^{-3}
RFX4⟷RFX2	RFX	RFX	8.59×10^{-3}
THRA, RXRB⟷NR2C2	Nuclear receptor	Nuclear receptor	8.59×10^{-3}
ESR1⟷SP3	Nuclear receptor	C2H2 ZF	8.59×10^{-3}
ESR1⟷KLF5, KLF2, KLF4	Nuclear receptor	C2H2 ZF	4.27×10^{-3}
ESR1⟷ZBTB1	Nuclear receptor	C2H2 ZF	1.05×10^{-3}
TFAP4⟷CTCF	bHLH	C2H2 ZF	1.77×10^{-2}
PURA⟷ESR2, ESRRA	Unknown	Nuclear receptor	1.77×10^{-2}
KLF8⟷CTCF	C2H2 ZF	C2H2 ZF	4.93×10^{-4}
HEY2⟷HAND1	bHLH	bHLH	3.61×10^{-2}
KLF15⟷CTCF	C2H2 ZF	C2H2 ZF	4.93×10^{-4}
SP8, KLF14, KLF16⟷CTCF	C2H2 ZF	C2H2 ZF	6.06×10^{-7}

Table S16. Significant matches with HIPPIE database inferred by SATORI deep with Pre-loaded CNN and Entropy Loss using Attn-Attr scores on Human Promoters Dataset

TF Interaction	TF1 Family	TF2 Family	P-value
MBD2⟷ESR1	MBD	Nuclear receptor	5.53×10^{-3}
NR0B1⟷ESR1	Unknown	Nuclear receptor	5.53×10^{-3}
NR0B1⟷PPARG	Unknown	Nuclear receptor	2.18×10^{-2}
SP2, SP1⟷ESR1	C2H2 ZF	Nuclear receptor	5.53×10^{-3}
SP2, SP1⟷ETS1, SMARCC2	C2H2 ZF	Ets, Myb/SANT	1.39×10^{-3}
NFIA, NFIB, NFIX⟷SMAD2	SMAD	SMAD	2.18×10^{-2}
NFIA, NFIB, NFIX⟷MYF5, MYOD1	SMAD	bHLH	4.31×10^{-2}
JUN⟷PURA	bZIP	Unknown	4.31×10^{-2}
WT1⟷NR2C2	C2H2 ZF	Nuclear receptor	3.63×10^{-5}
SMAD2⟷ESR1	SMAD	Nuclear receptor	4.31×10^{-2}
TFCP2⟷PBX3, NFYC	Grainyhead	Homeodomain, Unknown	1.09×10^{-2}
SMAD3, SMAD4⟷ETS1, SMARCC2	SMAD	Ets, Myb/SANT	1.09×10^{-2}
SMAD3, SMAD4⟷CTCF	SMAD	C2H2 ZF	2.80×10^{-3}
RFX4⟷RFX2	RFX	RFX	2.18×10^{-2}
THRA, RXRB⟷NR2C2	Nuclear receptor	Nuclear receptor	1.09×10^{-2}
ESR1⟷SP3	Nuclear receptor	C2H2 ZF	1.09×10^{-2}
ESR1⟷KLF5, KLF2, KLF4	Nuclear receptor	C2H2 ZF	5.53×10^{-3}
ESR1⟷ZBTB1	Nuclear receptor	C2H2 ZF	1.39×10^{-3}
TFAP4⟷CTCF	bHLH	C2H2 ZF	2.18×10^{-2}
ETS1, SMARCC2⟷KDM2B	Ets, Myb/SANT	CxxC	1.55×10^{-4}
ETS1, SMARCC2⟷ETS2	Ets, Myb/SANT	Ets	6.59×10^{-4}
PURA⟷ESR2, ESRRA	Unknown	Nuclear receptor	2.18×10^{-2}
KLF8⟷CTCF	C2H2 ZF	C2H2 ZF	6.59×10^{-4}
KLF15⟷CTCF	C2H2 ZF	C2H2 ZF	6.59×10^{-4}
SP8, KLF14, KLF16⟷CTCF	C2H2 ZF	C2H2 ZF	9.43×10^{-7}

REFERENCES
